# Biological Effects of BET Inhibition by OTX015 (MK-8628) and JQ1 in NPM1-Mutated (NPM1c) Acute Myeloid Leukemia (AML)

**DOI:** 10.3390/biomedicines9111704

**Published:** 2021-11-17

**Authors:** Hanane Djamai, Jeannig Berrou, Mélanie Dupont, Marie-Magdelaine Coudé, Marc Delord, Emmanuelle Clappier, Alice Marceau-Renaut, Anna Kaci, Emmanuel Raffoux, Raphaël Itzykson, Caroline Berthier, Hsin-Chieh Wu, Rita Hleihel, Ali Bazarbachi, Hugues de Thé, André Baruchel, Claude Gardin, Hervé Dombret, Thorsten Braun

**Affiliations:** 1 Laboratoire de Transfert des Leucémies, URP-3518, Institut de Recherche Saint Louis, Université de Paris, 75010 Paris, France; djamai.hanane@univ-paris-diderot.fr (H.D.); jeannig.berrou@univ-paris-diderot.fr (J.B.); melanie.dupont@univ-paris-diderot.fr (M.D.); marie-magdelaine.coude@lab-cerba.com (M.-M.C.); anna.kaci@u-paris.fr (A.K.); emmanuel.raffoux@aphp.fr (E.R.); andre.baruchel@aphp.fr (A.B.); claude.gardin@aphp.fr (C.G.); herve.dombret@aphp.fr (H.D.); 2 Laboratory of Hematology, Hôpital Saint-Louis, AP-HP, Université de Paris, 75010 Paris, France; emmanuelle.clappier@aphp.fr; 3 Bioinformatics, Institut de Recherche Saint Louis, Université de Paris, 75010 Paris, France; marc.delord@univ-paris-diderot.fr; 4 Laboratory of Hematology, CHRU Lille, 59037 Lille, France; Alice.MARCEAU@CHRU-LILLE.FR; 5 Leukemia Unit, Hematology Department, Hôpital Saint-Louis, AP-HP, Université de Paris, 75010 Paris, France; raphael.itzykson@aphp.fr; 6 INSERM U944—CNRS UMR7212, Institut de Recherche Saint Louis, Université de Paris, 75010 Paris, France; caroline.berthier@college-de-france.fr (C.B.); hsin-chieh.wu@college-de-france.fr (H.-C.W.); hugues.dethe@inserm.fr (H.d.T.); 7 Department of Anatomy, Cell Biology and Physiological Sciences, Faculty of Medicine, American University of Beirut, Beirut P.O. Box 113-6044, Lebanon; rh150@aub.edu.lb (R.H.); bazarbac@aub.edu.lb (A.B.); 8 Department of Pediatric Hemato-Immunology, Hôpital Robert Debré, AP-HP, Université de Paris, 75010 Paris, France; 9 Hematology Department, Hôpital Avicenne, AP-HP, Université de Paris, 93000 Bobigny, France

**Keywords:** BET inhibitors, OTX015 (MK-8628), JQ1, ATRA, ATO, *HOX* genes, NPM1, AML, differentiation

## Abstract

BET inhibitors (BETi) including OTX015 (MK-8628) and JQ1 demonstrated antileukemic activity including *NPM1c* AML cells. Nevertheless, the biological consequences of BETi in *NPM1c* AML were not fully investigated. Even if of better prognosis AML patients with *NPM1c* may relapse and treatment remains difficult. Differentiation-based therapy by all trans retinoic acid (ATRA) combined with arsenic trioxide (ATO) demonstrated activity in *NPM1c* AML. We found that BETi, similar to ATO + ATRA, induced differentiation and apoptosis which was *TP53* independent in the *NPM1c* cell line OCI-AML3 and primary cells. Furthermore, BETi induced proteasome-dependent degradation of NPM1c. BETi degraded NPM1c in the cytosol while BRD4 is degraded in the nucleus which suggests that restoration of the NPM1/BRD4 equilibrium in the nucleus of *NPM1c* cells is essential for the efficacy of BETi. While ATO + ATRA had significant biological activity in *NPM1c* IMS-M2 cell line, those cells were resistant to BETi. Gene profiling revealed that IMS-M2 cells probably resist to BETi by upregulation of LSC pathways independently of the downregulation of a core BET-responsive transcriptional program. ATO + ATRA downregulated a *NPM1c* specific *HOX* gene signature while anti-leukemic effects of BETi appear *HOX* gene independent. Our preclinical results encourage clinical testing of BETi in *NPM1c* AML patients.

## 1. Introduction

Acute myeloid leukemia (AML) is a heterogeneous group of genetically complex hematopoietic stem cell malignancies and is caused by oncogenic events including gene mutations and cytogenetic abnormalities conferring maturation arrest with clonal expansion of abnormal hematopoietic progenitors leading to bone marrow failure [1,2]. Outcome generally remains poor with overall survival ranging from 20–50% for different subgroups, depending on the age and feasibility of intensive treatment strategies [3]. Cytogenetic and genetic alterations classify AML into different risk categories [4,5]. Patients with normal karyotype belong to the intermediate risk category and their prognosis is determined by specific genetic alterations, including the Nucleophosmin 1 mutation (*NPM1c*) and FMS-like tyrosine kinase-3 (*FLT3*) internal tandem duplication (ITD), whereas isolated *NPM1c* confers generally rather good prognosis [4,6].

The *NPM1* gene (*NPMwt*) encodes a nucleolar shuttling protein, which regulates stabilization of the p14Arf tumor suppressor protein, P53 activation, ribosome biogenesis, and control of centrosome duplication [7]. About 30% of AML patients express a heterozygous *NPM1c* of which more than 80% are type A mutants [8]. NPM1c is delocalized to the cytoplasm compared to NPM1wt which is localized in the nucleus [9]. Nuclear localization of NPM1 is due to a nucleolar localization signal of which at least one is lost in *NPM1c* cells leading to its export in the cytosol and in consequence to the loss of its tumor suppressive function and subsequent expression of *HOX* genes [10,11]. The importance of *HOX* genes for *NPM1c* AML maintenance was demonstrated as specific loss of NPM1c from the cytoplasm leading to the downregulation of HOX genes and differentiation in *NPM1c* AML [11]. It was recently demonstrated that the activation of HOXBLINC, a *HOXB* locus-associated long non-coding RNA, is a critical downstream mediator of *NPM1c*-associated leukemic transcription program and leukemogenesis [12].

Standard treatment of AML patients with normal karyotype and *NPM1c* consists in the use of intensive chemotherapy (IC) leading to 5-year overall survival up to 60% without HSCT [3]. Nevertheless, relapse is frequent and there persists an unmet clinical need for novel treatment options for patients ineligible for IC and/or HSCT. Novel therapeutic approaches for *NPM1c* AML were reported recently. Among those, it was shown that differentiation-based therapy by all trans retinoic acid (ATRA) and arsenic trioxide (ATO) induced proteasomal degradation of NPM1c leading to exclusive nuclear localization of NPM1wt, differentiation, growth arrest, and P53-dependent apoptosis in *NPM1c* cells [13,14]. Furthermore, ATO + ATRA exposure significantly reduced bone marrow blasts in relapsed/refractory AML patients bearing *NPM1c* [13].

The bromodomain (BRD) and extraterminal (BET) inhibitors (BETi) I-BET151, JQ1, and OTX015 (MK-8628) were shown to provide antileukemic effects in *NPM1c* leukemic cells [15,16]. The impaired control of epigenetic readers of BRD, including BRD2, BRD3, and BRD4 is crucial for oncogenic processes in hematologic malignancies [17,18,19]. NPM1wt interacts with BRD4 in the nucleus and HEXIM1 represses BRD4-mediated transcriptional elongation via conformational changes of P-TEFb [16,20,21]. If *NPM1* is mutated, BRD4 and NPM1 are dissociated activating aberrant transcriptional elongation, resulting in increased expression of a BET specific core transcriptional program containing critical regulators of leukemogenesis like *BCL2*, *C-MYC,* and *IRF8* which are *HOX* gene independent [16]. Treatment of *NPM1c* AML cells with BETi I-BET151 resulted in downregulation of this specific core transcriptional program and subsequent cell cycle arrest, induction of apoptosis, decreased proliferation, and I-BET151 prolonged survival in a *NPM1c* mouse model [16]. OTX015 (MK-8628) was the first in class inhibitor to be tested in a phase I dose-escalating multicenter trial in hematologic diseases (NCT01713582). Response rates in acute leukemias were modest including 5 responses among 36 patients (13.9%) enrolled with 1 responder having AML with *NPM1c* [22,23]. It was recently demonstrated that BETi-resistant cells yield a leukemic stem cell (LSC)- like signature including upregulation of the Wnt/Beta-catenin pathway [24,25].

Based on those data, we investigated BETi compared to ATO + ATRA in *NPM1c* cell lines and patient samples to investigate their potential for further clinical development. BETi induced apoptosis, differentiation, and proteasome-dependent degradation of the NPM1c protein while the *NPM1c* cell line IMS-M2 appeared resistant to BETi possibly due to intrinsic upregulation of LSC pathways including the JAK/STAT, MAPK, and Wnt/Beta-catenin pathways. Furthermore, disappearance of aberrant NPM1c in the cytosol is generally induced by BETi in *NPM1c* cells and patient-derived blast cells. While ATO + ATRA downregulated a *NPM1c* specific *HOX* gene signature anti-leukemic effects of BETi appeared *HOX* gene independent. Thus, BETi have significant biologic effects in *NPM1c* cells and patient samples and clinical testing of these novel agents in *NPM1c* AML patients should be considered.

## 2. Materials and Methods

### 2.1. Cell Lines and Selection of Primary Patient Cells

OCI-AML3 (*NPM1c* type A and other mutations see Table 1) and K562 (*BCR-ABL+*) cell lines were purchased from Deutsche Sammlung von Mikroorganismen und Zellkulturen (DSMZ, Braunschweig, Germany). IMS-M2 cell line (ETV6-NTRK3 fusion, *NPM1c* type A, and other mutations see Table 1) was a kind gift from Pr Hugues de Thé. Cells were cultured in RPMI 1640 (Life Technologies, Courtaboeuf, France) supplemented with 10% heat-inactivated fetal bovine serum, 2 mM l-glutamine, 100 IU/mL penicillin, 100 μg/mL streptomycin and HEPES, at 37 °C with 5% CO_2_ as described elsewhere [15].

Mononuclear cells from the bone marrow (BM) of selected *NPM1c*-mutated AML patients were isolated by separation medium for lymphocytes (Eurobio, Courtaboeuf, France) as previously described [15]. Primary cells were maintained in IMDM (Life Technologies, Courtaboeuf, France) supplemented with 10% heat-inactivated fetal bovine serum, 2 mM l-glutamine, 100 IU/mL penicillin, and 100 μg/mL streptomycin without growth factors, at 37 °C with 5% CO_2_ as reported formerly [15]. Patients provided informed consent prior to BM aspiration at diagnosis, according to the Declaration of Helsinki. Approval for this study was obtained from the local institutional review board of the CHU Saint Louis, Paris (France). One patient with relapsed *NPM1c* AML was treated in a phase I dose escalating multicenter trial in hematologic diseases (NCT01713582) and patient BM cells were obtained at day 1 and 22 during the first treatment cycle. The patient provided informed consent prior to BM aspiration at diagnosis, according to the Declaration of Helsinki and the study was approved by the local institutional review boards of CHU Lille (France), CHU Saint Louis, Paris (France), CHU Lyon Sud (France), Institut Paoli Calmettes, Marseille (France), CHU Toulouse (France), Princess Margaret Cancer Center, Toronto (Canada) and Royal Marsden Hospital, London (UK) for all patients included in the trial NCT01713582, approved date: 24 October 2012 [22].

OTX015 (MK-8628) was purchased from CliniSciences (Nanterre, France) and JQ1 were purchased from tebu-bio (Le Perray en Yvelines, France). ATRA (all-trans retinoic acid) and ATO (Arsenic trioxide) diarsenic trioxide were purchased from Sigma (Saint Quentin Fallavier, France). Bortezomib (PS-341) were purchased from Euromedex (Souffelweyersheim, France). All compounds were dissolved in dimethyl sulfoxide (DMSO) purchased from Euromedex (Souffelweyersheim, France) and stored at −20 °C, except ATO that was dissolved in water and stored at 4 °C. Aliquots were thawed and used immediately for serial dilution in culture media. Control cells were incubated with 0.1% DMSO.

### 2.2. ApoTox-Glo^TM^ and Apoptosis Assessment

For the *ApoTox-Glo^TM^* Triplex Assay from Promega (Charbonnières Les Bains, France), cells were seeded in 48-well plates at 0.2 × 10^6^/mL and treated with a range of ATO-ATRA concentrations from 0.009 µM to 5 µM, for OTX015 (MK-8628) and JQ1 from 0.00038 µM to 100 µM for 72 h. Cells were transferred to 96-well plates and incubated with viability/cytotoxicity reagent containing both GF-AFC substrate and bis-AAF-R110 in the dark at 37 °C for 30 min. The fluorescence was measured at 400Ex/505Em for the viability and 485Ex/520Em for the cytotoxicity. The cells were incubated with Caspase-Glo^®^ 3/7 reagent for 30 min at room temperature and the luminescence was measured. Three independent experiments were run for each cell line and untreated cells were used as negative controls. The half maximal inhibitory concentration (IC50) values were calculated with Prism^®^ v6 software (GraphPad Software LLC, La Jolla, CA, USA). For apoptosis analysis, 1 × 10^6^ cells derived from patients or cell lines were resuspended in 1 mL culture medium and treated for 48 h and 72 h with either 500 nM OTX015 (MK-8628), 500 nM JQ1, 1 µM ATRA + 1 µM ATO or 0.1% DMSO. Apoptotic cells were detected using a CytoFLEX flow cytometer (Beckman Coulter, Villepinte, France). Cells were stained with 5 µg/mL PI and Annexin-V-FITC (Becton Dickinson, Le Pont de Claix, France) according to the manufacturer’s instructions for 15 min at room temperature. Apoptotic cells were defined as Annexin V + with or without PI uptake. Apoptosis was determined by cytofluorometric analysis and analyzed with the FlowJo flow cytometry software (FlowJo LLC, Ashland, OR, USA).

### 2.3. CRSPR-Cas9 TP53 Kock Out

To CRISPR out P53, gRNA targeting human P53 (5′CCATTGTTCAATATCGTCCG), negative control (CGTTAATCGCGTATAATACGGTTTTAGAGCTATGCT), and recombinant Cas9 protein was synthesized from IDT to form Alt-R CRISPR/Cas9 RNP. OCI-AML3 cells were transiently transfected with Alt-R CRISPR/Cas9 RNP by using Nucleofector kit T (Amaxa) and applied program number X-01 in the nucleofector device (Lonza). The stable CRISPR knockout clones undergo serial dilution for single-cell separation. The DNA was extracted from these clones and the region surrounding the Cas9 cutting site was PCR amplified for sequencing.

### 2.4. Differentiation Assessment

Morphologic differentiation of OCI-AML3, IMS-M2, K562 or patient cells was determined by CD11b expression and morphologic assessment of cells stained with May-Grünwald-Giemsa. Cells were treated for 48 h and 72 h with either 500 nM OTX015 (MK-8628), 500 nM JQ1, 1 µM ATRA + 1 µM ATO, or 0.1% DMSO followed by flow cytometric detection of the differentiation marker CD11b Alexa Fluor^®^ 488 (#557701, Becton Dickinson, Le Pont de Claix, France) using a CytoFLEX flow cytometer (Beckman Coulter, Villepinte, France) and analyzed with the FlowJo flow cytometry software (FlowJo LLC, Ashland, OR, USA). For morphologic analysis, cytospin preparations (Cytospin 4, Fisher Scientific, Illkirch, France) of OCI-AML3, IMS-M2, K562 or patient cells treated for 48 h by OTX015 (MK-8628) 500 nM, JQ1 500 nM, ATRA 1 µM + ATO 1 µM, or 0.1% DMSO were stained with May-Grünwald Giemsa solution and examined under light microscopy with a Zeiss microscope (Carl Zeiss, Marly le Roi, France) with a X40 objective.

### 2.5. Immunoblotting

Protein was extracted from 5 × 10^6^ cells exposed to either 500 nM OTX015 (MK-8628), 500 nM JQ1, 1 µM ATRA, 1 or 3 µM ATO, or 0.1% DMSO; 30 μg of protein was loaded on SDS-polyacrylamide gels using 4–15% gradient gels (#4568084, Bio-Rad, Marnes-La-Coquette, France) and transferred to nitrocellulose membranes (#1704158, Bio-Rad, Marnes-La-Coquette, France) using a Mini Trans-Blot Electrophoretic Transfer cell (Bio-Rad, Marnes-La-Coquette, France). Membranes were blocked with LI-COR blocking buffer (#927-40000, Eurobio, Courtaboeuf, France) and incubated with the primary antibody overnight at 4 °C: rabbit anti-NPM1m (#PA1-46356, Life Technologies, Courtaboeuf, France), mouse anti-NPM1total (#H00004869-M01), mouse anti-TP53 (#sc-126) both of CliniSciences, Nanterre, France. Rabbit anti-PARP (#9542), rabbit anti-cleaved caspase 3 (#9661), mouse anti-vinculin (#sc-73614) were purchased from Ozyme, Saint-Cyr-l’École, France, and mouse anti-GAPDH (#398600) from Life Technologies, Courtaboeuf, France. Goat anti rabbit HRP or goat anti mouse HRP secondary antibodies (#1706515 and # 1706516, Bio-Rad, Marnes-La-Coquette, France), were incubated for 1 h at room temperature. Bands were visualized using the ChemiDoc Touch (Bio-Rad, Marnes-La-Coquette, France).

### 2.6. Separation of Nuclear and Cytosolic Extracts

Cytoplasmic and nuclear protein were extracted from 5 × 10^6^ OCI-AML3 cells exposed to OTX015 (MK-8628), JQ1, ATO + ATRA or 0.1% DMSO with NE-PER Nuclear and Cytoplasmic kit (#78833, Life Technologies, Courtaboeuf, France). Proteins thus obtained were concentrated with Amicon Ultra centrifugal filters, Ultracel 30 K and 10 K (#UFC503096 and #UFC501096, Merck Millipore, St. Quentin Fallavier, France). Thirty microgrammes of cytoplasmic proteins and 5–10 µg of nuclear proteins were loaded on SDS-polyacrylamide gels using 4–15% gradient gels (Bio-Rad, Marnes-La-Coquette, France) and transferred to nitrocellulose membranes using a Mini Trans-Blot Electrophoretic Transfer cell (Bio-Rad, Marnes-La-Coquette, France). Membranes were blocked with LI-COR blocking buffer and incubated with the primary antibody overnight at 4 °C: anti-NPM1m (#ab65816, Abcam, Paris, France), anti-NPM1total (#H00004869-M01, CliniSciences, Nanterre, France), anti-BRD4 (#ab128874, Abcam, Paris, France), anti-tubulin (#ab176560, Abcam, Paris, France), or anti-lamin (#ab16048, Abcam, Paris, France). Secondary antibodies were goat anti rabbit HRP or goat anti mouse HRP (Bio-Rad, Marnes-La-Coquette, France), incubated for 1 h at room temperature. Bands were visualized using the ChemiDoc Touch (Bio-Rad, Marnes-La-Coquette, France).

### 2.7. Immunofluorescence

OCI-AML3, IMS-M2, and K562 or patient cells were fixed for 20 min with methanol at −20 °C, then cytospined on polylysine-L slides (#045797, Dominique Dutscher, Brumath, France), permeabilized using Triton X-100 0.5% for 15 min and blocked for 1 h with 3% BSA in DPBS (pH 7.4). Cells were incubated with a mouse anti NPM1 antibody recognizing its wild type and mutated form (#sc-47725, CliniSciences, Nanterre, France) 1 h at room temperature, slides were then washed and incubated for 1 h at room temperature with a secondary antibody goat anti-mouse IgG-coupled Alexa 488 fluorochrome (#A-11017, Life Technologies, Courtaboeuf, France). Nuclei were counterstained with DAPI (#H-1200, Eurobio, Courtaboeuf, France). Images were acquired by immunofluorescence microscopy on a Zeiss Axiovert microscope with a Plan-Apochromat X40 N.A.1.4 oil immersion objective using the Axiovison software v4.2 (Carl Zeiss, Marly le Roi, France).

### 2.8. In Situ Proximity Ligation Assays (Duolink^®^)

OCI-AML3 cells were fixed in methanol onto glass coverslips by cytospin. Protein-protein interactions were visualized using the Duolink^®^ in situ proximity ligation assay (PLA) system from Sigma (Saint Quentin Fallavier, France), using an anti-NPMwt + c antibody (#sc-47725, CliniSciences).

### 2.9. Microarray

Gene expression profile was performed as described previously [15]: untreated OCI-AML3 and IMS-M2 cells were compared to treated cells (exposure for 24 h either 500 nM OTX015 (MK-8628), 500 nM JQ1, 1 µM ATRA, 1 µM ATO, 1 µM ATRA + 1 µM ATO, or 0.1% DMSO). After extraction of total RNA, 500 ng of RNA of each sample was processed using WT PLUS Amplification and Labeling Kit according to the manufacturer’s instructions. Analysis was performed with the GeneChip Human Transcriptome Array HTA 2.0 Array (Affymetrix^®^, Life Technologies, Courtaboeuf, France). We generated each sample in triplicates (*n* = 21).

We analyzed differential gene expression using the Bioconductor limma library on annotated coding transcripts (*n* = 32,670) and metabolic pathways analysis was performed using the GSEA v2.0 software with 1000 gene set permutations.

### 2.10. Targeting Sequencing Using NGS

Targeting sequencing of all exons of 66 genes recurrently involved in myeloid hematological malignancies was performed using a customized SureSelect™ (Agilent) capture panel (List of genes below). Libraries were prepared from 200 ng of genomic DNA from leukemia cells according to manufacturer’s instructions and sequenced on NextSeq 550™ using Illumina reagents. The mean depth was 995× and the coverage at a minimum depth of 200× was 97%.

Data analysis was performed using an in-house pipeline using GATK, Varscan and Pindel and VISCAP tools. Interpretation of variants was performed with the help of human genome databases, COSMIC database, prediction algorithms (including SIFT and PolyPhen) and literature review. List of genes: ASXL1, ASXL2, ATRX, BCOR, BCORL1, BRAF, BRCA2, BRCC3, CALR, CBL, CEBPA, CREBBP, CSF3R, CTCF, CUX1, DDX41, D NMT3A, EP300, ERCC6L2, ETNK1, ETV6, EZH2, FLT3, GATA2, HRAS, IDH1, IDH2, IRF1, JAK2, KDM5A, KDM6A, KIT, KMT2A/MLL, KMT2D/MLL2, KRAS, MECOM, MPL, MYC, NF1, NPM1, NRAS, PHF6, PPM1D, PRPF8, PTEN, PTPN11, RAD21, RIT1, RUNX1, SAMD9, SAMD9L, SBDS, SETBP1, SF1, SF3B1, SMC1A, SMC3, SRP72, SRSF2, STAG2, TET2, TP53, U2AF1, U2AF2, WT1, ZRSR2.

## 3. Results

### 3.1. Antileukemic Effects of BETi in NPM1c-Mutated Cell Lines

**Figure 1:**
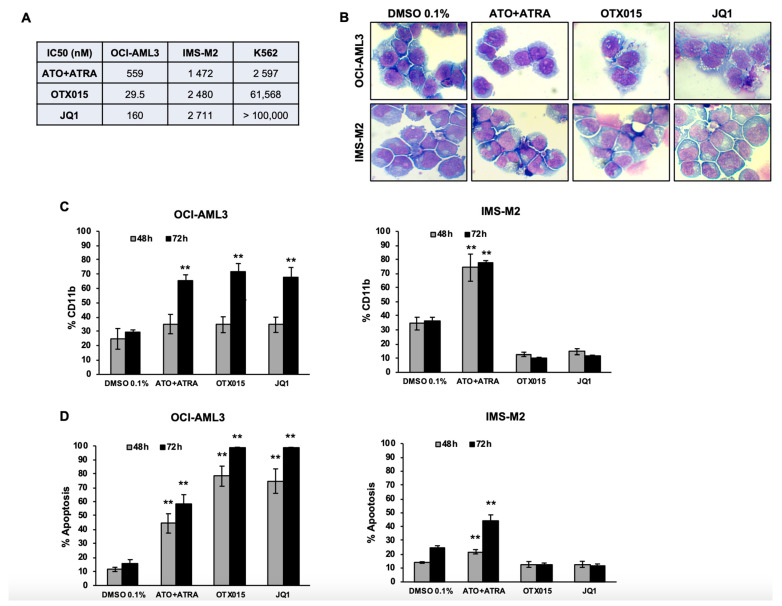
IC50, differentiation and apoptosis induced by BETi compared to ATO + ATRA in NPM1c and K562 cell lines. (**A**) OCI-AML3, IMS-M2, and K562 were exposed for 72 h to increasing doses of indicated drugs, and ApoTox-Glo^TM^ assays were performed to determine IC50 of the indicated cell lines. (**B**) Cell morphology of OCI-AML3 and IMS-M2 after treatment either with 0.1% DMSO, 1000 nM ATO + 1000 nM ATRA, 500 nM OTX015 (MK-8628), or 500 nM JQ1 was studied at 48 h by May-Grünwald-Giemsa staining after cytospin. (**C**) The expression of the differentiation marker CD11b was detected by flowcytometry using a human CD11b-specific mouse monoclonal antibody on OCI-AML3 and IMS-M2 cells treated for 48–72 h either with 0.1% DMSO, 1000 nM ATO + 1000 nM ATRA, 500 nM OTX015 (MK-8628), or 500 nM JQ1. (**D**) OCI-AML3 or IMS-M2 cells were exposed 48–72 h either to 0.1% DMSO, 1000 nM ATO + 1000 nM ATRA, 500 nM OTX015 (MK-8628), or 500 nM JQ1. Apoptosis was detected with annexin V and PI by using flow cytometry. Apoptotic cells were defined as annexin V + with or without PI uptake. Results are shown as mean ± SEM from duplicates of three independent experiments. ** represents *p* ≤ 0.01.

We investigated the effects of BETi OTX015 (MK-8628) and JQ1 compared to ATO + ATRA for their ability to affect cell viability, differentiation, and to induce apoptosis in the NPM1c cell lines OCI-AML3, IMS-M2, and K562. The OCI-AML3 cell line was particularly sensitive to BETi (IC50 29.5 nM for OTX015 (MK-8628) and 160 nM for JQ1) (Figure 1A, Supplementary Figure S1). For IMS-M2 cells and the NPM1wt cell line K562, IC50 was >1 μM for OTX015 (MK-8628), JQ1 and ATO + ATRA. According to recently published results of ATO + ATRA to induce differentiation in NPM1c cells, we were interested whether BETi may have the same effects [13]. When exposed to OTX015 (MK-8628) or JQ1 OCI-AML3 cells displayed morphological signs of differentiation similar to ATO + ATRA exposure, namely reduction in cytoplasmic basophilia and an increased nuclear lobulation observed in nonapoptotic cells (Figure 1B). Differentiation was observed for IMS-M2 cells after ATO + ATRA exposure while no signs of differentiation were observed after treatment with BETi in this cell line. In line, treatment of OCI-AML3 cells with BETi and ATO + ATRA-induced expression of the differentiation marker CD11b which was only observed in IMS-M2 cells after exposure to ATO + ATRA (Figure 1C, Supplementary Figure S2). No differentiation was detected in the resistant NPMwt cell line K562 (Supplementary Figure S3). BETi induced higher levels of apoptosis as detected by annexin V and PI compared to ATO + ATRA exposure in OCI-AML3 cells while IMS-M2 cells showed no signs of apoptosis after exposure to OTX015 (MK-8628) or JQ1, while ATO + ATRA induced significant Annexin V exposure and PI incorporation in those cells (Figure 1D).

### 3.2. Apoptosis Triggered by BETi in NPM1c-Mutated Cells Is Independent from TP53

**Figure 2:**
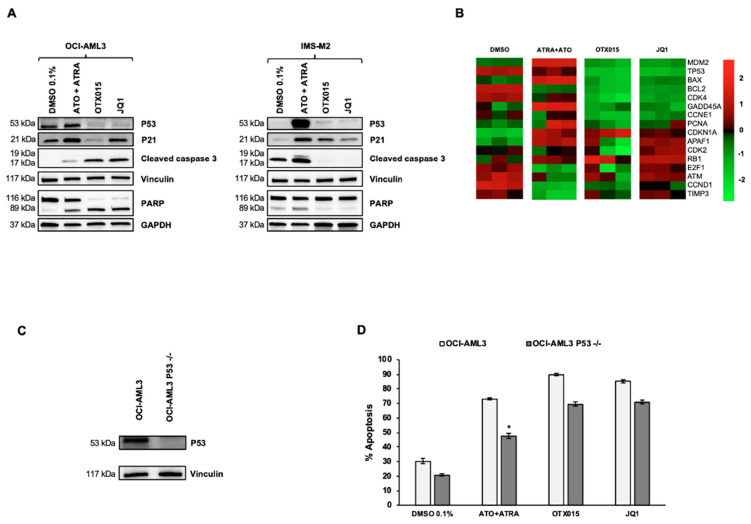
Treatment with OTX015 (MK-8628) and JQ1 induces P53-independent apoptosis in OCI-AML3 cells. (**A**) Western blot showing P53, P21, caspase 3, and PARP protein changes in OCI-AML3 or IMS-M2 cells treated with similar doses of indicated drugs for 72 h. Vinculin was used as loading control for P53, P21, and caspase 3 and GAPDH for PARP. One representative experiment out of three is shown for WB experiments. Experiments were performed as triplicates. (**B**) Heatmap displaying expression of genes related to the TP53 pathway in OCI-AML3 cells treated for 72 h with 0.1% DMSO, 1000 nM ATO + 1000 nM ATRA, 500 nM OTX015 and 500 nM JQ1. The experiment was conducted on biological triplicates. (**C**) OCI-AML3 cells were transfected by electroporation with Alt-R CRISPR/Cas9 RNP. Knock out of TP53 was controlled with Western blot detecting P53 protein for indicated cells. Vinculin was used as loading control. One representative experiment out of three is shown. (**D**) OCI-AML3 knocked out for TP53 by CRISPR was exposed to 500 nM OTX015 (MK-8628), 500 nM JQ1, 1000 nM ATO + 1000 nM ATRA, or 0.1% DMSO for 72 h. Apoptosis was detected with annexin V and PI by using flow cytometry. Results are shown as mean ± SEM from duplicates of three independent experiments. Statistically significant differences were calculated from medians compared by the Mann–Whitney U test.* represents *p* ≤ 0.05.

As expected, P53 expression was observed upon treatment of OCI-AML3 and IMS-M2 cells with ATO + ATRA but interestingly no P53 expression was observed after BETi exposure for those cells (Figure 2A). In line, treatment of both cell lines with ATO + ATRA induced subsequent caspase 3 and PARP cleavage. For BETi sensitive OCI-AML3 cells we observed also caspase 3 and PARP cleavage upon BETi exposure while no signs of cleavage of those proteins were observed with BETi treatment of IMS-M2 cells. Furthermore, we investigated expression of the P53 downstream target P21. Interestingly, we observed induction of P21 by ATO + ATRA and BETi in both NPM1c cell lines without induction of caspase 3 and PARP cleavage in IMS-M2 cells (Figure 2A). TP53 is an important general executor of apoptosis in various models and it was shown that ATO + ATRA-induced apoptosis in NPM1c cells depends on TP53 [13,14,26]. We performed gene expression profiling after treatment with BETi and ATO + ATRA for 24 h (Figure 2B). In NPM1c OCI-AML3 cells, ATO + ATRA induced upregulation of genes of the TP53-dependent pathway (including TP53, MDM2, BAX), while treatment with BETi leads to the downregulation of this pathway. Furthermore, we performed knock out experiments with CRISPR/Cas9 for TP53 in OCI-AML3 cells. CRISPR/Cas9 transfection for TP53 efficiently reduced P53 expression (Figure 2C). Knock out of TP53 reduced significantly sensitivity of OCI-AML3 cells to ATO + ATRA but not to BETi (Figure 2D).

### 3.3. OTX015 (MK-8628) and JQ1 Induce Proteasome-Dependent Degradation of the NPM1c Oncoprotein

**Figure 3:**
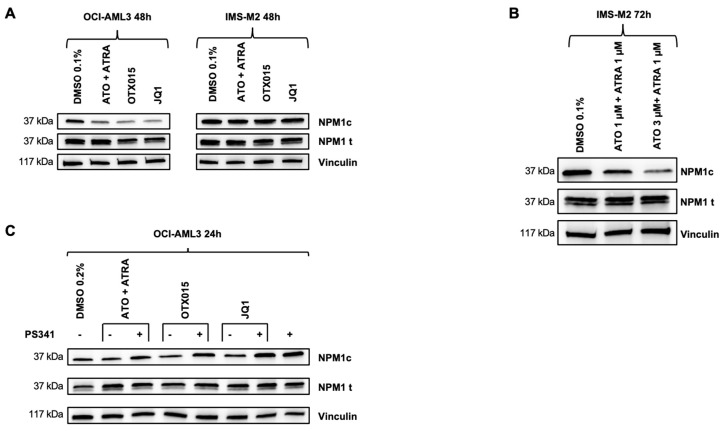
Effects of BETi on NPM1c/NPMwt protein expression. (**A**) OCI-AML3 cells were exposed 48 h either to 0.1% DMSO, 500 nM OTX015 (MK-8628), 500 nM JQ1, or 1000 nM ATO + 1000 nM ATRA. Western blot showing protein expression of NPM1c oncoprotein and total NPM1 by using appropriate antibodies were performed. Vinculin was used as loading control. One representative experiment out of three is shown. (**B**) IMS-M2 cells were exposed 72 h either to 0.1% DMSO, 1000 nM ATO + 1000 nM ATRA, or 3000 nM ATO + 1000 nM ATRA. Western blot showing protein expression of NPM1c oncoprotein and total NPM1 by using appropriate antibodies were performed. Vinculin was used as loading control. One representative experiment out of three is shown. (**C**) OCI-AML3 cells were exposed 24 h either to 0.2% DMSO, 500 nM OTX015 (MK-8628), 500 nM JQ1, or 1000 nM ATO + 1000 nM ATRA in the presence or absence of 10 nM of the proteasome inhibitor PS341 (bortezomib). Western blot showing protein changes of NPM1c oncoprotein and total NPM1 protein by using appropriate antibodies was performed. Vinculin was used as loading control. One representative experiment out of three is shown.

ATRA and to a lesser extend ATO and their association were shown to degrade specifically the NPM1c oncoprotein in a proteasome-dependent manner [13,14]. Interestingly, BETi OTX015 (MK-8628) and JQ1 treatment also decreased NPM1c protein levels in NPM1c OCI-AML3 cells similar to ATO + ATRA (Figure 3A). NPM1c degradation upon ATO + ATRA treatment of IMS-M2 cells was more obvious after increasing doses of ATO (3 μM) and no degradation of NPM1c was seen after exposure to OTX015 (MK-8628) and JQ1 in IMS-M2 cells (Figure 3A,B). The degradation of NPM1c was most obvious after 48 h of treatment. Addition of the proteasome inhibitor PS-341 (bortezomib) to ATO + ATRA, OTX015 (MK-8628), and JQ1 reduced NPM1c degradation at 24 h for BETi and at 48 h for ATO + ATRA indicating that NPM1c degradation is at least in part dependent on proteasome activity (Figure 3C, Supplementary Figure S4A,B). Protein interaction assays showed ubiquitination of NPM1c upon treatment with ATO + ATRA and BETi (Supplementary Figure S4C). At later time points NPM1c protein was degraded due to late cytotoxic effects of drug combinations with bortezomib (data not shown).

### 3.4. Effects of BETi on NPM and BRD4 in OCI-AML3 Cells

**Figure 4:**
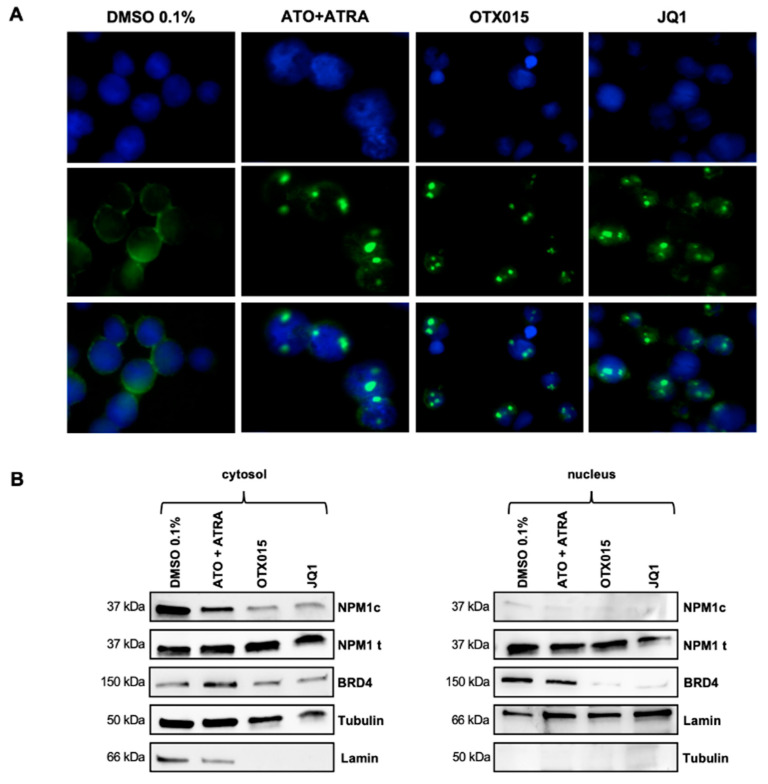
Intracellular shuttling of NPM1 and BRD4 after BETi treatment of OCI-AML3 cells. (**A**) Confocal immunofluorescence microscopy for cellular localization of NPM1 (wt + c) was performed after 48 h exposure either to 0.1% DMSO, 1000 nM ATO + 1000 nM ATRA, 500 nM OTX015 (MK-8628), or 500 nM JQ1. Cells were stained with an antibody-recognizing NPM (wt + c) (green) and nuclei are labeled blue by DAPI. One representative experiment out of three is shown. (**B**) OCI-AML3 cells were exposed 48 h either to 500 nM OTX015 (MK-8628), 500 nM JQ1, 1000 nM ATO + 1000 nM ATRA, or 0.1% DMSO and nuclear and cytosolic proteins were extracted. Western blot showing protein changes of NPM1c oncoprotein, total NPM1 protein, and BRD4 by using appropriate antibodies were performed. Tubulin and lamin were used as appropriate loading controls and proper separation controls (not shown) of the cytosol and the nucleus respectively. One representative experiment out of three is shown.

In NPM1c AML some amount of oligomeric NPM1 (wt + c) is aberrantly localized from the nucleus to the cytosol leading to diffuse cytoplasmic stain [9,14,27]. It was recently reported that treatment with ATO + ATRA abolished localization of NPM1wt in the cytosol after degradation of NPM1c [11,13,14]. We could show that treatment of the NPM1c cell lines OCI-AML3 and IMS-M2 with OTX015 (MK-8628) and JQ1 had similar effects. Upon treatment with BETi, NPM1 increased in a typical NPMwt pattern in the nucleus while NPM1wt and NPM1c disappeared from the cytosol as compared to controls and similar to ATO + ATRA treatment as described previously and demonstrated here by immunofluorescence (Figure 4A, Supplementary Figure S5). Those effects were not observed in the NPM1wt cell line K562 (Supplementary Figure S5). It is thought that NPM1wt associates with BRD4 inhibiting its uncontrolled transcriptional activity in NPM1-unmutated cells [16]. Dislocation and dissociation of NPM1wt from BRD4 in NPM1c-mutated AML may increase aberrant transcriptional activity of BRD4 in the nucleus. Thus, we were interested to study modifications of BRD4 and NPM1c in the nucleus and in the cytosol. After treatment of OCI-AML3 cells with OTX015 (MK-8628), JQ1, and ATO + ATRA, extracts of the nucleus and the cytosol were obtained and proteins were studied by Western blot (Figure 4B). In the cytosol, OTX015 (MK-8628) and JQ1 as well as ATO + ATRA decreased NPM1c protein levels while BRD4 protein was not decreased. In the nucleus, OTX015 (MK-8628) and JQ1 decreased BRD4 protein levels. No NPM1c protein was found in nuclear extracts. Altogether these data suggest that degradation of the NPM1c oncoprotein in the cytosol and BRD4 degradation in the nucleus contribute to BET inhibitor-triggered biological effects in NPM1c AML.

### 3.5. Biologic Effects Induced by OTX015 (MK-8628) in NPM1c Patient Samples

**Figure 5:**
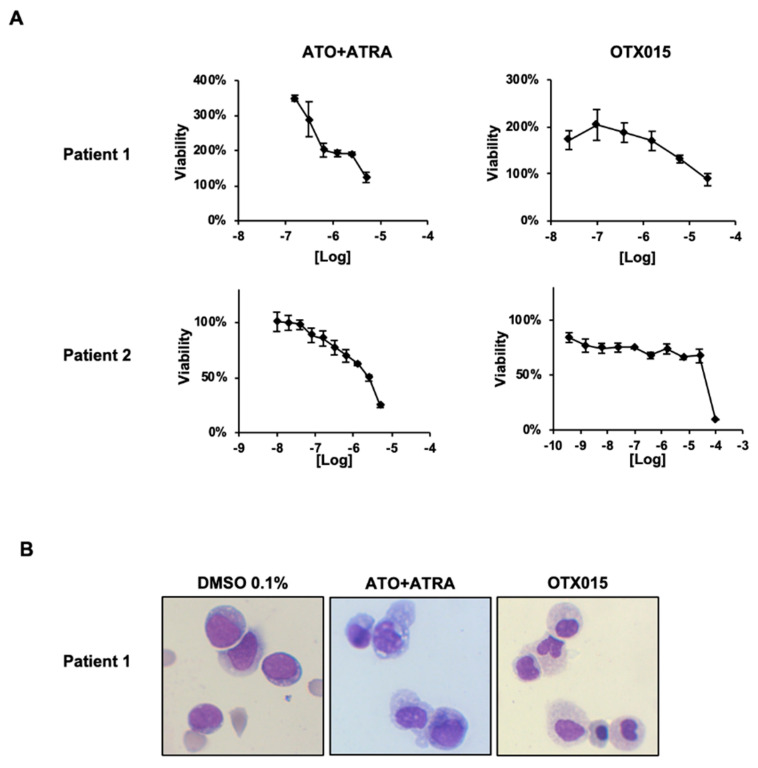
Effects of BETi in NPM1c patient-derived bone marrow blast cells. Primary leukemic bone marrow blast cells were obtained from two indicated NPM1c + AML patients. (**A**) For two patients, blast cells were exposed ex vivo for 72 h to increasing doses of indicated drugs and ApoTox-Glo^TM^ assays were performed to determine viability of the patient cells. (**B**) Cell morphology of patient derived blast cells after treatment either with 0.1% DMSO, 1000 nM ATO + 1000 nM AT-RA, 500 nM or OTX015 (MK-8628) was studied at 48 h by May-Grünwald-Giemsa staining after cytospin.

**Figure 6:**
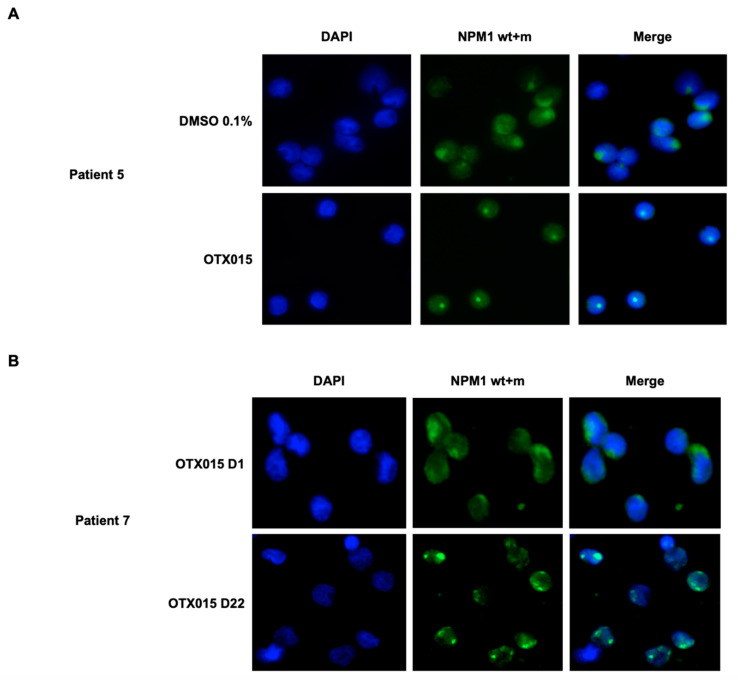
Ex vivo and in vivo treatment of patient-derived NPM1c bone marrow blast cells with OTX015 (MK-8628)-induced disappearance of NPM1c in the cytosol. Primary leukemic bone marrow blast cells were obtained from three NPM1c + AML patients. (**A**) For two patients blast cells were exposed ex vivo for 48 h to 0.1% DMSO or 500 nM OTX015 (MK-8628). One representative patient is shown. (**B**) For a patient treated in the phase I study in vivo by OTX015 (MK-8628) 120 mg once daily, bone marrow blasts were obtained on day 1 (80% BM blasts) and day 22 (60% BM blasts). Cells were analyzed by confocal immunofluorescence microscopy for cellular localization of NPM1 (wt + c). Cells were stained with an antibody-recognizing NPM1 (wt + c) (green) and nuclei are labeled blue by DAPI.

We obtained patient-derived bone marrow samples from four patients (Table 2, Patient #1–4). Bone marrow blasts were exposed to OTX015 (MK-8628) and ATO + ATRA. We tested only OTX015 (MK-8628) which is the clinically relevant drug as JQ1 cannot be administered in vivo for patients and as patient samples were limited in number of cells. In representative patients we could observe loss of blast cell viability which varied for patients and drug type (Figure 5A). OTX015 (MK-8628) as well as ATO + ATRA induced differentiation in blast cells treated ex vivo as detected by morphologic analysis (Figure 5B). Furthermore, bone marrow samples from patients (*n* = 2) with NPM1c were treated ex vivo by OTX015 (MK-8628). In line with the observations in the NPM1c cell lines, exposure to OTX015 (MK-8628) leads to disappearance of NPM1c in the cytosol as shown in 1 representative patient by immunofluorescence (Patient #5; Patient #6 data not shown; Figure 6A; Table 2). We took advantage of the phase I dose-escalating multicenter trial in hematologic diseases (NCT01713582) including 1 patient with relapsing NPM1c AML in our center. The patient received 120 mg of OTX015 (MK-8628) once daily and bone marrow examination was performed on day 1 and day 22 and bone marrow blasts were subjected immediately to IF staining without further exposure to OTX015 (MK-8628) ex vivo. On day 1 of treatment with OTX015 (MK-8628) the NPM1c was detected in the cytosol while on day 22 NPM1c disappeared from the cytosol with the patient having stable disease at this time point (Figure 6B, Patient #7, Table 2). The patient remained stable for two cycles of treatment until progression. Taken together we demonstrate that BETi induce apoptosis, differentiation, and disappearance of NPM1c in the cytosol in NPM1c primary cells.

### 3.6. Resistance of IMS-M2 Cells to BETi Is Mediated by a Specific Transcriptional Program

**Figure 7:**
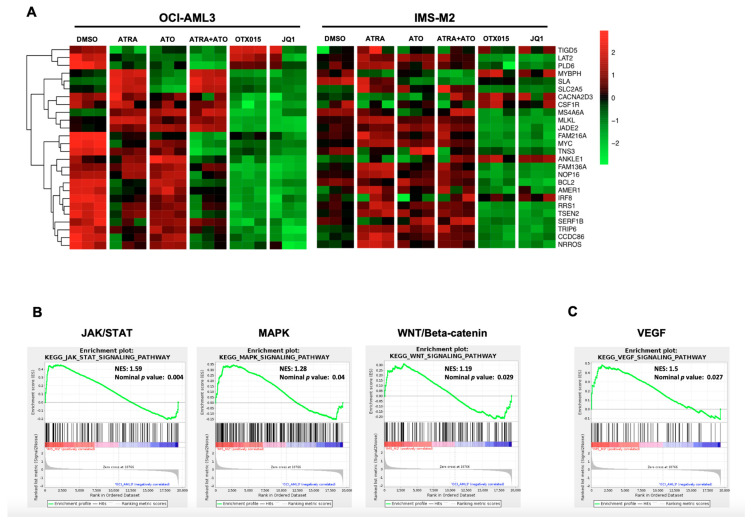
BRD signature and molecular profiles in NPM1c OCI-AML3 and IMS-M2 cell lines. GeneChip Human Transcriptome Array HTA 2.0 (Affymetrix^®^) was performed for OCI-AML3 and IMS-M2 cell lines. Experiments were performed as triplicates. (**A**) Heatmap of the specific BRD signature [16] of cells either treated with 0.1% DMSO, 1000 nM ATRA, 1000 nM ATO, 1000 nM ATO + 1000 nM ATRA, 500 nM OTX015 (MK-8628), or 500 nM JQ1. (**B**) GSEA plots of IMS-M2 cells compared to OCI-AML3 cells showing significantly upregulated gene pathways in IMS-M2 cells (KEGG_JAK_STAT_signaling_pathway, KEGG_MAPK_signaling_pathway, KEGG_WNT_signaling_pathway). (**C**) GSEA plot of IMS-M2 cells compared to OCI-AML3 cells showing significantly upregulated KEGG_VEGF_signaling pathway in IMS-M2 cells; NES, normalized enrichment score.

To investigate the sensitivity/resistance profile of NPM1c cell lines OCI-AML3 and IMS-M2 to BETi, we performed microarray analysis of these cell lines without and upon treatment with OTX015 (MK-8628) or JQ1 compared to ATO + ATRA. It was recently shown that BETi alter a specific BET core signature in AML which is highly expressed in NPM1c AML and downregulation of genes within this program, such as BCL2 and C-MYC, leads to the induction of apoptosis and cell cycle arrest [16]. Thus, we were interested if OCI-AML3 or IMS-M2 cell lines differ in expression of this program to explain differences in sensitivity to BETi. Both cell lines expressed this BET core signature associated with NPM1c AML (Figure 7A). Treatment of OTX015 (MK-8628) and JQ1 strongly repressed this program including BCL2 and MYC (Figure 7A, Supplementary Figure S6). BETi resistance emerges from leukemic stem cells (LSC) and deregulated pathways in LSC including MAPK, JAK-STAT, and Wnt/Beta-catenin signaling pathways [24,25]. We could show that these pathways are significantly upregulated in BETi-resistant IMS-M2 cells (Figure 7B). Furthermore, we found significant upregulation of the VEGF pathway also reported to be associated with BETi resistance (Figure 7C) [24]. To determine differences in gene mutations in OCI-AML3 and IMS-M2 cell lines which may be potentially implicated in BETi resistance of IMS-M2 cells, we performed NGS for mutated genes. Both cell lines had the same NPM1c type A mutation which was associated with a D NMT3A mutation (Table 1). The cell lines differed in additional mutations with OCI-AML3 cells having a NRAS mutation and IMS-M2 having a MECOM and a CTCF mutation. Furthermore, IMS-M2 cells are known to carry the ETV6-NTRK3 gene fusion [28]. Our results indicate that BETi resistance in IMS-M2 cells could be mediated by the upregulation of LSC pathways.

### 3.7. Effects of BETi Treatment on HOX Gene Signatures

**Figure 8:**
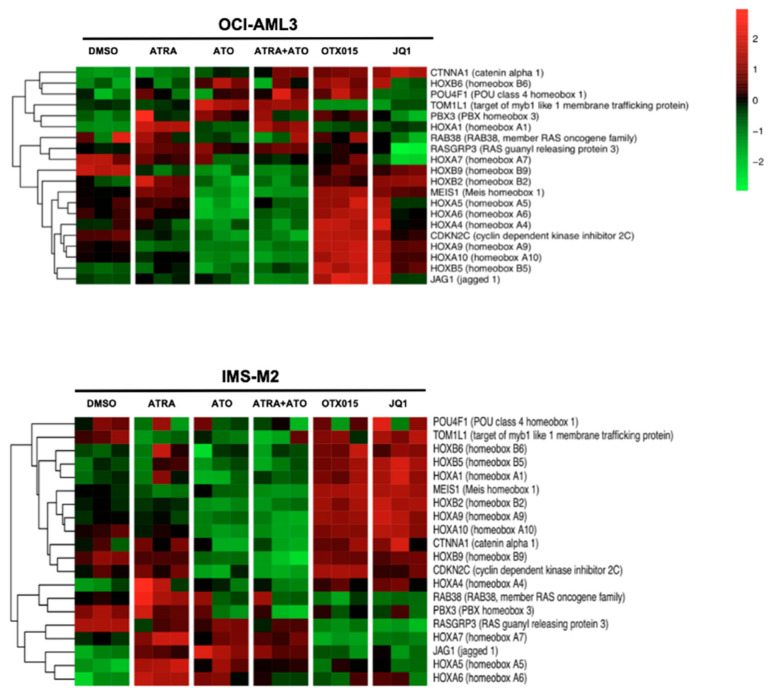
HOX gene cluster signatures in NPM1c OCI-AML3 and IMS-M2 cell lines after treatment with BETi and ATO + ATRA. GeneChip Human Transcriptome Array HTA 2.0 (Affymetrix^®^) was performed for OCI-AML3 and IMS-M2 cell lines. Experiments were performed as triplicates. Heatmap of the specific HOX signature [10] of cells either treated with 0.1% DMSO, 1000 nM ATRA, 1000 nM ATO, 1000 nM ATO + 1000 nM ATRA, 500 nM OTX015 (MK-8628), or 500 nM JQ1.

It is thought that BETi affect the BET core signature in a HOX gene-independent manner [16]. Nevertheless, NPM1c AML cells display a unique gene expression profile, characterized by high expression of multiple HOXA and HOXB clusters [10]. ATO with or without ATRA treatment induced downregulation of this HOX gene signature in OCI-AML3 and IMS-M2 cell lines (Figure 8). In contrast, OTX015 (MK-8628) and JQ1 regulated differently HOX gene clusters. While BETi lead generally to HOX gene upregulation in NPM1c cell lines, OTX015 (MK-8628) induced upregulation of HOXA genes (HOXA5, HOXA6, and HOXA7) in OCI-AML3 cells and downregulation in BETi-resistant IMS-M2 cells. Upregulation of HOXB genes (i.e., HOXB2, HOXB5, HOXB6, and HOXB9) was observed in both cell lines (Figure 8).

## 4. Discussion

About 30–40% of AML patients bear *NPM1c* leading to the expression of an oncoprotein driving leukemogenesis in this subtype [9]. Even if not fully elucidated, *NPM1c* is an AML founding genetic lesion representing a potential target for therapeutic intervention. Even if of rather good prognosis many patients with *NPM1c* AML relapse and novel treatment approaches are needed.

Two recent studies demonstrated potent biological effects of ATRA and ATO particularly in combination in *NPM1c* OCI-AML3 cells, patient-derived blast cells and patients [13,14]. Those effects include apoptosis induction, differentiation, proteasome-dependent degradation of the NPM1c oncoprotein in the cytosol, NPMwt nucleolar localization and blast clearance in relapsed/refractory patients treated in a compassionate setting [13]. We compared biological effects of two BETi OTX015 (MK-8628) and JQ1 in *NPM1c* cells as preclinical data suggested that the *NPM1c* AML subtype may be particularly sensitive to this treatment approach [15,16]. It was shown that if *NPM1* is mutated, transcriptional repression of BRD4 by NPM1 is lost due to dissociation of both proteins [16]. This results in increased expression of specific BET core signature including oncogenic *c-MYC* and *BCL2* [16]. Exposure of OCI-AML3 to the pan BET inhibitor I-BET151 resulted in the downregulation of *BCL2* and *c-MYC* and subsequent induction of apoptosis and decreased proliferation. Furthermore, significant activity of I-BET was found in a *NPM1c* mouse model [16]. We have already demonstrated that OTX015 (MK-8628) and JQ1 induce apoptosis in OCI-AML3 cells and downregulation of *BCL2* and *c-MYC* [15]. Interestingly we found here that exposure to BETi induced differentiation, proteasome-dependent NPM1c degradation leading to disappearance of NPM1c in the cytosol and mainly nuclear localization of NPM1wt. Nevertheless, BETi triggered effects on *NPM1c* cells yielding some differences compared to ATO + ATRA treatment. ATO + ATRA-induced apoptosis is P53 dependent in *NPM1c* cells [13,14,26]. Similar to ATO + ATRA, BETi lead to caspase 3 and PARP cleavage. In OCI-AML3 cells, ATO + ATRA induced strong upregulation of genes of the *TP53*-dependent pathway (*BAX*/*GADD45)* while the anti-apoptotic gene *BCL2* was downregulated. In contrast, treatment with BETi lead to strong downregulation of the *TP53*-dependent pathway also including *BCL2* which is a biomarker of BETi treatment [15,17]. OTX015 (MK-8628) and JQ1 did not induce P53 protein expression and TP53 knock out by CRISPR left apoptosis rather unaffected upon exposure to OTX015 (MK-8628) and JQ1 suggesting that apoptosis induced by BET inhibitors is at least partially *TP53* independent. This should be investigated in *TP53*-mutated AML subtypes with complex karyotypes as *TP53* mutations occur only rarely upon clonal evolution in relapsed *NPM1c* patients [29]. BETi may overcome treatment resistance due to *TP53*-independent apoptosis mechanisms. Interestingly, BETi induced differentiation comparable to ATO + ATRA, as detected by CD11b surface expression and by morphologic analysis in OCI-AML3 cells indicating that BETi are also capable to induce differentiation in *NPM1c* AML as it has been shown in MLL-driven AML formerly [19]. Furthermore, it would be interesting to study in the future the precise mechanisms of these drugs on differentiation and to design comprehensive drug combinations. It was recently shown that *NPM1c* abrogates monocyte and granulocyte terminal differentiation by disrupting the PU.1/CEBPA/RUNX1 interplay [30]. Especially combinations with the hypomethylating agent decitabine could be of potential interest as it drives granulocytic differentiation.

It was demonstrated that ATO + ATRA degrade the NPM1c oncoprotein in *NPM1c* cells and this degradation is at least in part dependent on proteasome activity [13]. Nevertheless, the precise mechanisms of NPM1c protein ubiquitination and degradation are unknown. It is thought that upon degradation, NPM1c-NPMwt oligomerization is disrupted and NPM1wt accumulates mainly in the nucleus where it exerts again its tumor-suppressive activity leading to differentiation and apoptosis [16,27]. Interestingly, we could show that BETi also lead to proteasome-dependent degradation of NPM1c in the cytosol and to main localization of NPM1 in the nucleus and it seems that this degradation is a common downstream effect of different treatment approaches. As shown by Dawson et al. [16] NPMwt-NPMwt oligomers shuttle from the nucleus to the cytosol. In *NPM1c* leukemic cells NPM1wt and NPM1c form an oligomer abrogating NPM1wt onco-suppressive activity. In NPM1wt cells, NPM1wt interacts with BRD4 in the nucleus and abrogates BRD4-mediated leukemogenic activity [16]. In our experiments BRD4 expression is suppressed upon BETi treatment in the nucleus. This suggests that restoration of the NPM1wt/BRD4 equilibrium in the nucleus of *NPM1c* cells is essential for the efficacy of BETi in *NPM1c* cells and differs from action induced by ATO + ATRA in *NPM1c* cells.

Intriguingly, the *NPM1c* cell line IMS-M2 is resistant to BETi treatment. Upon treatment with OTX015 (MK-8628) and JQ1 no apoptosis, differentiation, or NPM1c degradation could be observed. We performed gene profiling and BETi downregulate a BET-specific core gene signature in OCI-AML3 and IMS-M2 cells demonstrating that BETi trigger a common core gene signature reported for *NPM1c* AML. We demonstrate here with gene profiling analysis that IMS-M2 cells yield a resistance signature including upregulation of the LSC pathways MAPK, JAK-STAT, and Wnt/Beta-catenin signaling pathway. These pathways were shown to confer generally resistance to BETi in the LSC compartment and in BETi-resistant cell lines and cell clones [24,25]. We also found significant upregulation of the VEGF pathway also known to be associated with BETi resistance [24]. Our results indicate that *NPM1c* AML is heterogenous even if AML with NPM1 mutation is a distinct genetic entity in the revised World Health Organization classification [4]. Distinct co-mutations may impact on response to therapy and expression of different levels of LSC gene pathways may contribute to this heterogeneity. Two distinct subtypes within *NPM1c* AML patients were recently defined, which were labeled as primitive and committed based on the respective presence or absence of a stem cell signature which is in line with our results [31].

As shown by our microarray data *NPM1c* OCI-AML3 and IMS-M2 cell lines express the same HOX gene signature as published by Alcalay et al. for NPM1c patients [10]. ATO + ATRA downregulate the specific *NPM1c* HOX gene signature as it was recently reported for Selinexor treatment of *NPM1c* cells and this suggests a HOX gene-dependent pathway to trigger cell death and differentiation induced by these drugs in *NPM1c* cells [11]. On the other hand, as published by Dawson et al. BETi trigger a core BET-responsive transcriptional program containing BCL-2 and c-MYC [16]. We confirm that this program is also downregulated in OCI-AML3 and IMS-M2 cells without downregulation of the *NPM1c*-specific HOX program. This suggests a *HOX*-independent mechanism mediated by BCL-2 and c-MYC downregulation. Furthermore, in IMS-M2 cells resistance to BETi is mediated by upregulation of LSC pathways independently of the downregulation of the core BET-responsive transcriptional program.

As reported formerly for ATO + ATRA treatment, we could show the disappearance of NPM1c pattern in the cytosol in *NPM1c* cell lines and primary AML blasts upon treatment with BETi. Interestingly, NPM1c is not degraded in IMS-M2 by BETi cells but we could observe NPM1c disappearance in the cytosol in those cells. This indicates that BETi may interfere with NPM1c/NPM1wt oligomerization and that resistance mechanisms via deregulated gene pathways exert their activity up- or downstream in the tumor suppressive action of NPM1wt in IMS-M2 cells.

We could also demonstrate in vivo activity of OTX015 (MK-8628) in a patient with relapsing *NPM1c* AML treated in the phase I dose escalating multicenter trial in hematologic diseases (NCT01713582). On day 1 of treatment with 120 mg once daily OTX015 (MK-8628) the NPM1 protein (wt + c) was localized in the cytosol in bone marrow blast cells while on day 22 the NPM1c disappeared from the cytosol. This illustrates in vivo activity of BETi in an AML patient and recapitulates effects observed upon treatment with ATO + ATRA in relapsed/refractory *NPM1c* patients [13]. Furthermore, detection of NPM1 translocations in patient cells could be used as biomarker for clinical activity of different drugs in future clinical trials.

## 5. Conclusions

In conclusion, we could demonstrate that BETi induced apoptosis, differentiation, proteasomal degradation of NPM1c and its disappearance in the cytosol in *NPM1c* cells. These effects may differ according to *NPM1c* models with specific sensitivity and even resistance patterns due to heterogeneity of *NPM1c* AML. This delineates a comprehensive design of clinical trials with BETi in mono-or combination therapy for patients with *NPM1c* AML.

## Figures and Tables

**Table 1:** Mutations detected by NGS in OCI-AML3 and IMS-M2 cells.

Cell Line	Gene	Mutation	VAF%
OCI-AML3	NRAS	NM_002524:exon3:c.A182T:p.Q61L	100
	DNMT3A	NM_022552:exon23:c.C2644T:p.R882C	52
	NPM1	NM_002520:exon11:c.859_860insTCTG:p.L287fs	48
IMS-M2	DNMT3A	NM_022552:exon9:c.1079_1080insAA:p.N360fs	95
	NPM1	NM_002520:exon11:c.859_860insTCTG:p.L287fs	46
	MECOM	NM_001105078:exon7:c.A1745C:p.D582A	35
	CTCF	NM_006565:exon7:c.T1337C:p.I446T	7

**Table 2:** Characteristics of patients included in this study (* Provisional entity).

Patient No.	Age	Gender	WBC G/L	BM Blatsts%	Karyotype	NPM1 Status	Additional Mutations	FAB	WHO 2016 *
1	71	F	65	88	46XX; i (Xp)	MutA	IDH1 (R132C)	M1	AML NPM1c
2	72	F	75.8	93	46; XX	MutA	BCOR, TET2	M1	AML NPM1c
3	63	F	2.8	25	46; XX	MutA	CEBPA+/−, CSF3R, PTPN11, STAG2	M2	AML NPM1c
4	72	F	44.6	99	46; XX	MutA	TET2, FLT3-ITD	M1	AML NPM1c
5	47	F	43	76	46; XX; del (11q)	MutA	FLT3-ITD	M2	AML NPM1c
6	75	F	3.9	78	46; XX	MutA	FLT3-ITD, TET2	M1	AML NPM1c
7	76	M	7.2	43	46; XY	MutA	IDH1, NRAS, SF3B1	M2	AML NPM1c

## Data Availability

Our genomic data will be public in the GEO data base if the paper is accepted: The following secure token has been created to allow review of record GSE146362 while it remains in private status: kbyjkicojdqtfsj. Please note the following points: - This token allows anonymous, read-only access to GSE146362 and associated accessions while they are private; - Treat the token as you would a password and realize that the token provides access to GSE146362 to anyone who uses it; - we recommend you do not include the token anywhere except in secure email to journal editors; - If you want to revoke this access token, you can click the ‘Reviewer access’ button on your record which exposes the option to revoke this token. Send the following information to journal editors who will circulate to reviewers requiring access to your private data: To review GEO accession GSE146362: Go to https://www.ncbi.nlm.nih.gov/geo/query/acc.cgi?acc=GSE146362, Enter token kbyjkicojdqtfsj into the box.

## Supplementary Materials

The following are available online at https://www.mdpi.com/article/10.3390/biomedicines9111704/s1. Figure S1: Dose response curves for ATO + ATRA and BETi in NPM1c cell lines. Figure S2: Differentiation patterns of OCI-AML3 and IMS-M2 cell lines after exposure to BETi compared to ATO + ATRA. Figure S3: ATO + ATRA induced NPM1c protein degradation with or without bortezomib. Figure S4: Exposure to OTX015 (MK-8628) and JQ1-induced NPM1c disappearance in the cytosol of IMS-M2 cells. Figure S5: Significant downregulation of the BRD signature in NPM1c OCI-AML3 and IMS-M2 cell lines. Figure S6: Significant down regulation of the BRD signature in NPM1c OCI-AML3 and IMS-M2 cell lines.
