# Supplementary material for: Biological Effects of BET Inhibition by OTX015 (MK-8628) and JQ1 in NPM1-Mutated (NPM1c) Acute Myeloid Leukemia (AML)
Source: Biomedicines. 2021 Nov 17;9(11):1704. doi: 10.3390/biomedicines9111704 (PMC8615962; doi:10.3390/biomedicines9111704)
Supplement: Supplementary file 1 [file biomedicines-09-01704-s001.zip › Revised supplementary Figure Legends NPM Biomedicines.pdf]

Supplementary Figure Legends

Supplementary Figure S1: Dose response curves for ATO+ATRA and BETi in *NPM1c* cell lines and the *NPM1wt* cell line K562. Cell lines were exposed for 72h to increasing doses of indicated drugs and *ApoTox-Glo*^{TM} assays were performed to determine IC50 of the cell lines. X-axis: log of drug doses used, Y-axis: percent of cells. Values are means of 3. Three independent experiments were run for each cell line.

Supplementary Figure S2: Differentiation patterns of OCI-AML3 and IMS-M2 cell lines after exposure to BETi compared to ATO+ATRA. Histogramms of CD11b as detected by flowcytometry using a human CD11b-specific mouse monoclonal antibody on OCI-AML3 and IMS-M2 cells treated for 48-72h either with 0.1% DMSO, 1000nM ATO+1000nM ATRA, 500nM OTX015 (MK-8628) or 500nM JQ1. Results from one representative experiment of three independent experiments are shown.

Supplementary Figure S3: Exposure of the NPMwt K562 cell line to ATO+ATRA and BETi. (**A**) K562 cells were exposed 48-72h either to 0.1% DMSO, 1000nM ATO+1000nM ATRA, 500nM OTX015 (MK-8628) or 500nM JQ1. Apoptosis was detected with annexin V and PI by using flow cytometry. Apoptotic cells were defined as annexin V+ with or without PI uptake. Results are shown as mean +/- SEM from duplicates of three independent experiments. (**B**) The expression of the differentiation marker CD11b was detected by flowcytometry using a human CD11b-specific mouse monoclonal antibody on K562 cells treated for 48-72h either with 0.1% DMSO, 1000nM ATO+1000nM ATRA, 500nM OTX015 (MK-8628) or 500nM JQ1. Results are shown as mean +/- SEM from duplicates of three independent experiments. (**C**) Cell morphology of K562 after treatment either with 0.1% DMSO, 1000nM ATO+1000nM ATRA, 500nM OTX015 (MK-8628) or 500nM JQ1 was studied at 48h by May-Grünwald-Giemsa staining after cytospin. Experiments were performed as triplicates.

Supplementary Figure S4: ATO+ATRA and BETi induced NPM1c protein degradation with or without bortezomib. (**A**) Densitometry of protein changes of WB shown in **Figure 3 A**. (**B**) OCI-AML3 cells were exposed 48h either to 0.1% DMSO or 1000nM ATO+1000nM ATRA in the presence or absence of 10nM of the proteasome inhibitor PS341 (bortezomib). Western blot showing protein changes of NPM1c oncoprotein and total NPM1 protein by using appropriate antibodies were performed. Vinculin was used as loading control. One representative experiment out of three is shown. (**C**) Duolink® in situ proximity ligation assay performed in OCI-AML3 cells. As observed by IF microscopy NPM1c interacts in the cytosol

with ubiquitin (red dots) upon exposure with 1000nM ATO+1000nM ATRA, 500nM OTX015 (MK-8628) or 500nM JQ1. 0.1% DMSO induced no interaction with ubiquitin.

Supplementary Figure S5: Exposure to OTX015 (MK-8628) and JQ1 induced NPM1c disappearance in the cytosol of IMS-M2 cells. Confocal immunofluorescence microscopy for cellular localization of NPM1 (wt+c) was performed after 48h exposure either to 0.1% DMSO, 1000nM ATO+1000nM ATRA, 500nM OTX015 or 500nM JQ1. Cells were stained with an antibody recognizing NPM (wt+c) (green) and nuclei are labeled blue by DAPI. One representative experiment out of three is shown. K562 NPM1wt served as negative control.

Supplementary Figure S6: Significant down regulation of the BRD signature in *NPM1c* OCI-AML3 and IMS-M2 cell lines. GeneChip Human Transcriptome Array HTA 2.0 (Affymetrix®) was performed for OCI-AML3 and IMS-M2 cell lines. Experiments were performed as triplicates. GSEA plots of the specific *BRD* signature[14] of cells either treated with 500nM OTX015 or 500nM JQ1 compared with 0.1% DMSO; NES, normalized enrichment score.
