# Supplementary material for: Biological Effects of BET Inhibition by OTX015 (MK-8628) and JQ1 in NPM1-Mutated (NPM1c) Acute Myeloid Leukemia (AML)
Source: Biomedicines. 2021 Nov 17;9(11):1704. doi: 10.3390/biomedicines9111704 (PMC8615962; doi:10.3390/biomedicines9111704)
Supplement: Supplementary file 1 [file biomedicines-09-01704-s001.zip › Revised Supplementary Figures Biomedicines NPM1.pdf]

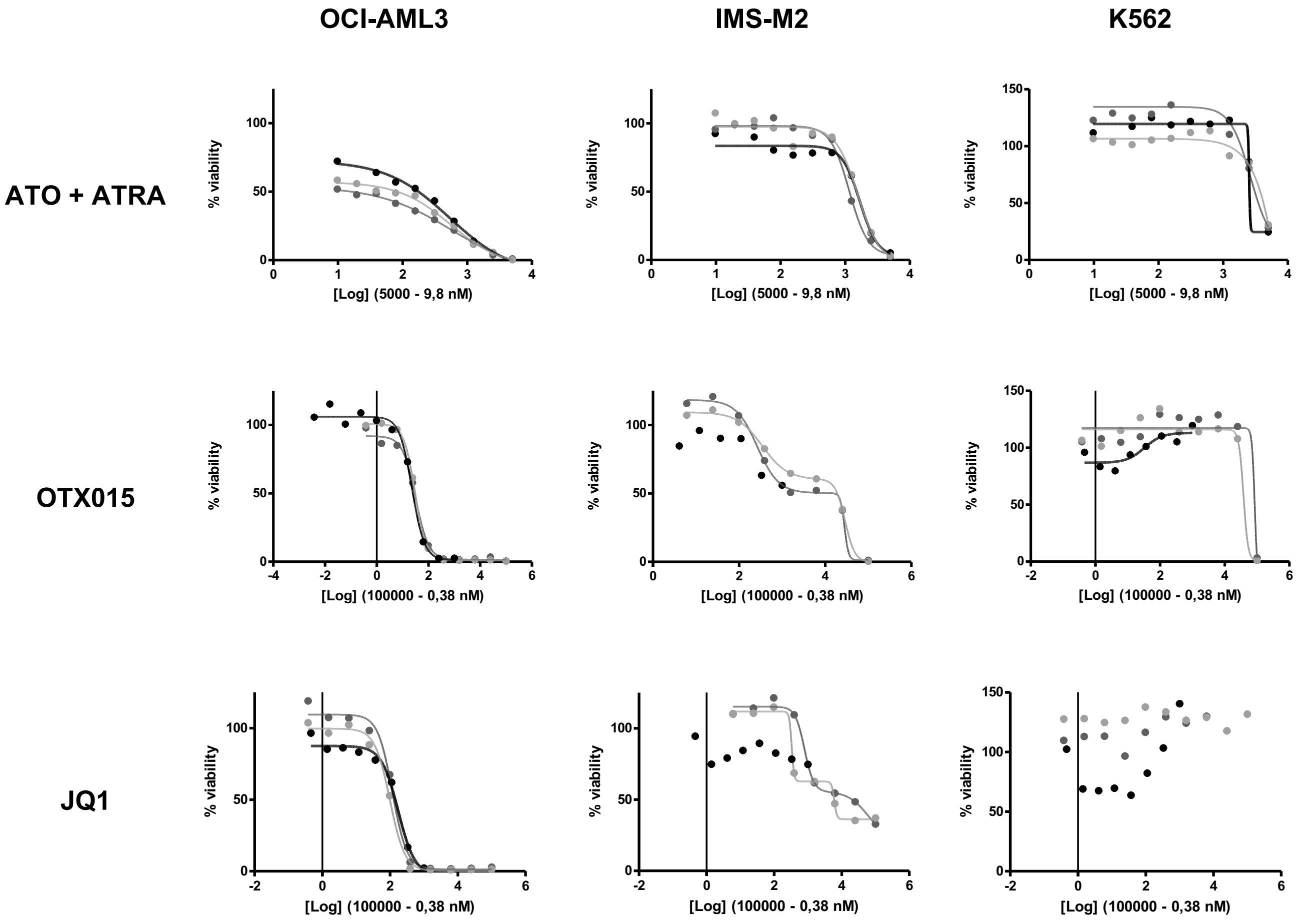

Supplementary Figure S1

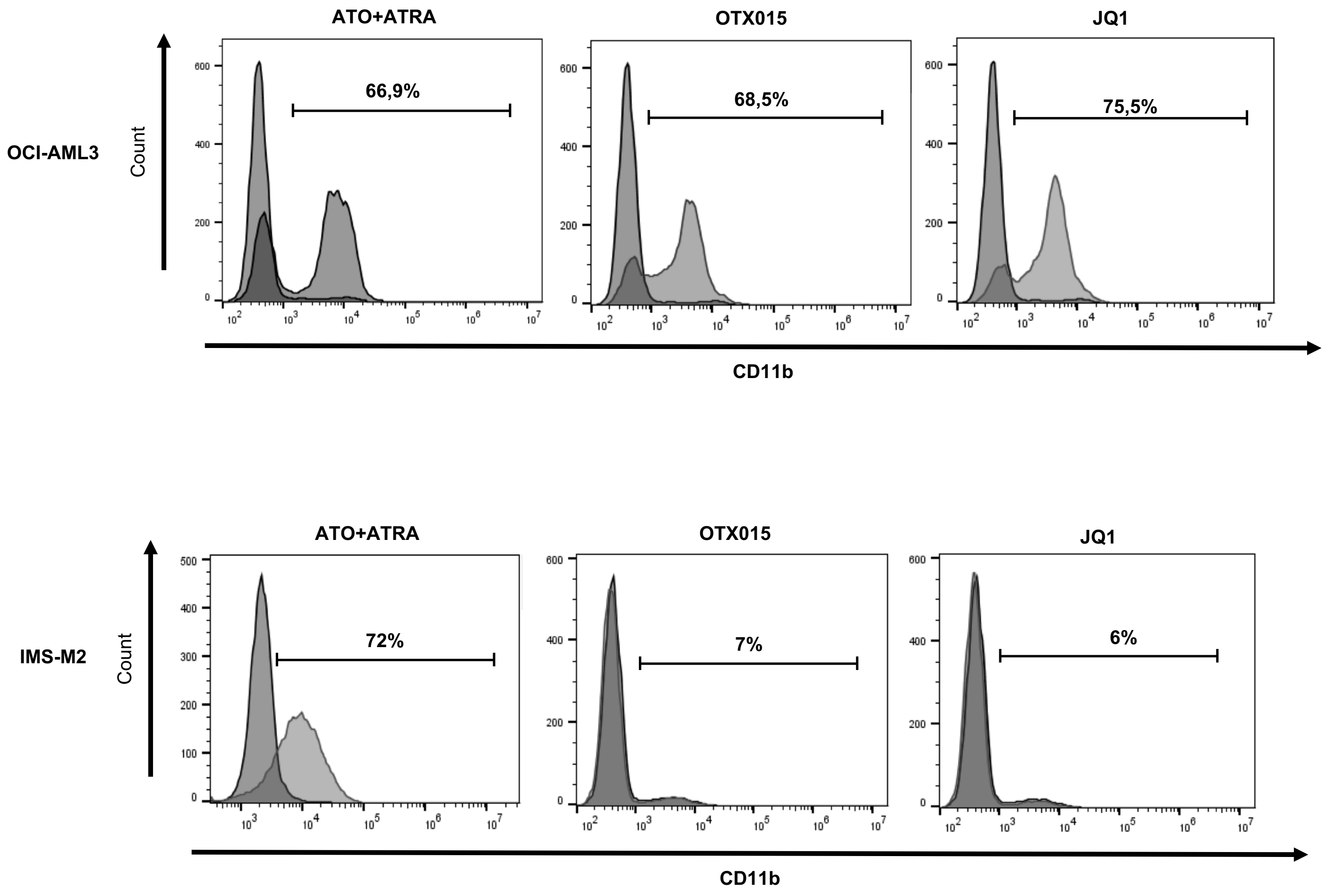

Supplementary Figure S2

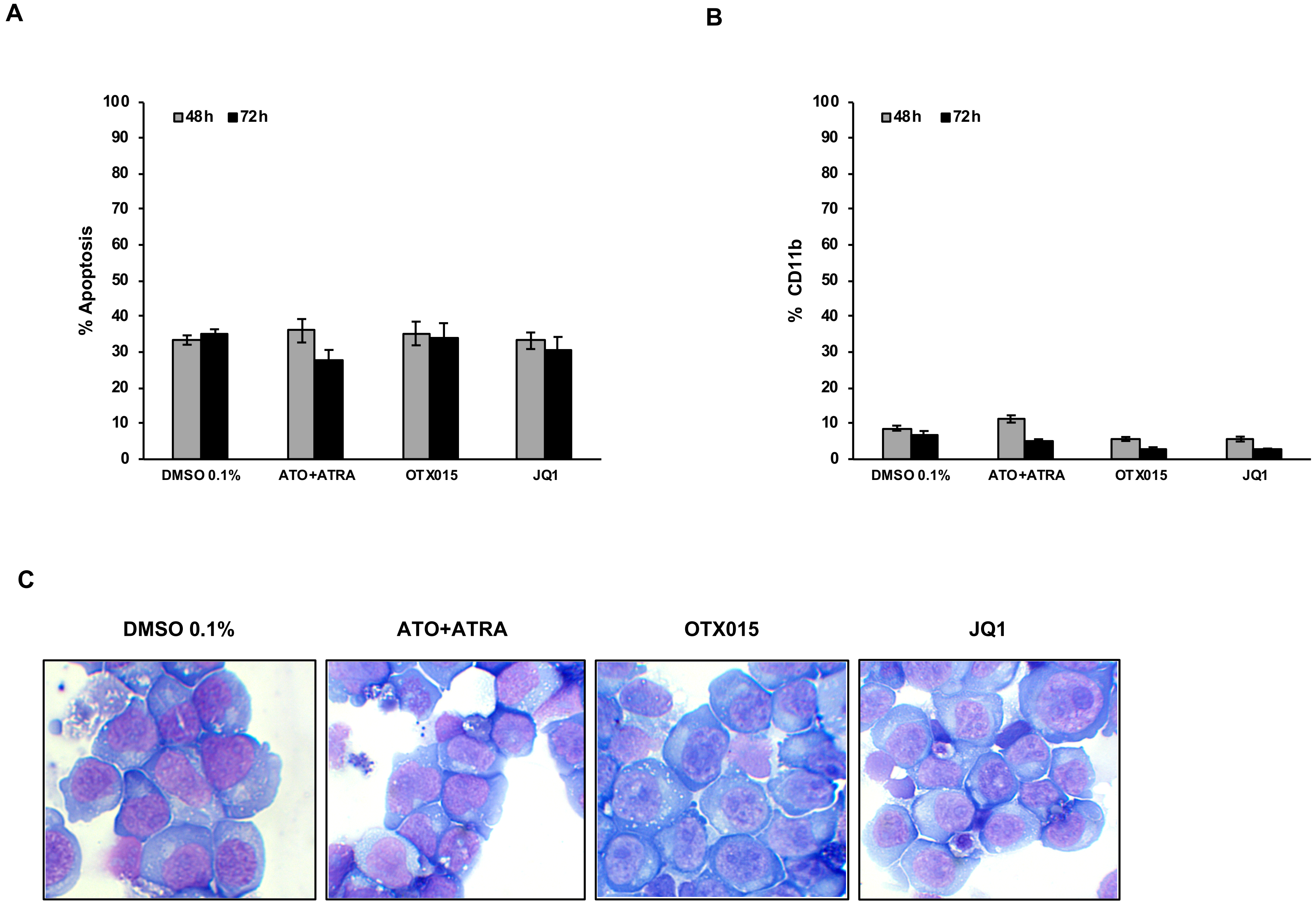

Supplementary Figure S3

A

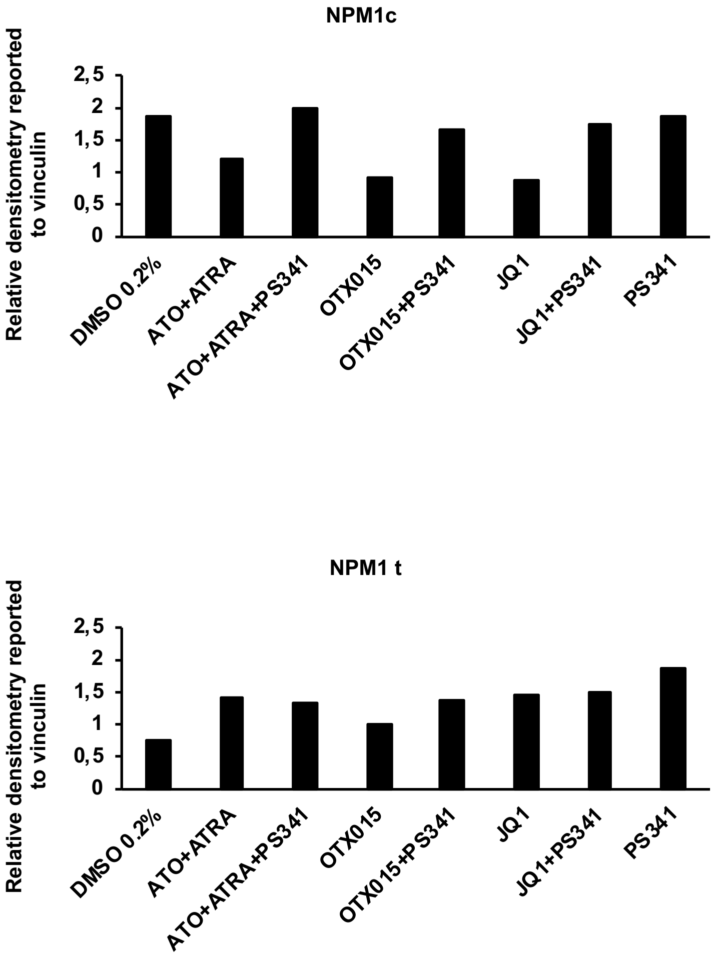

B

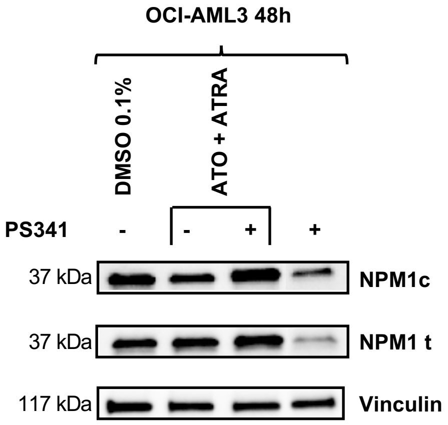

C

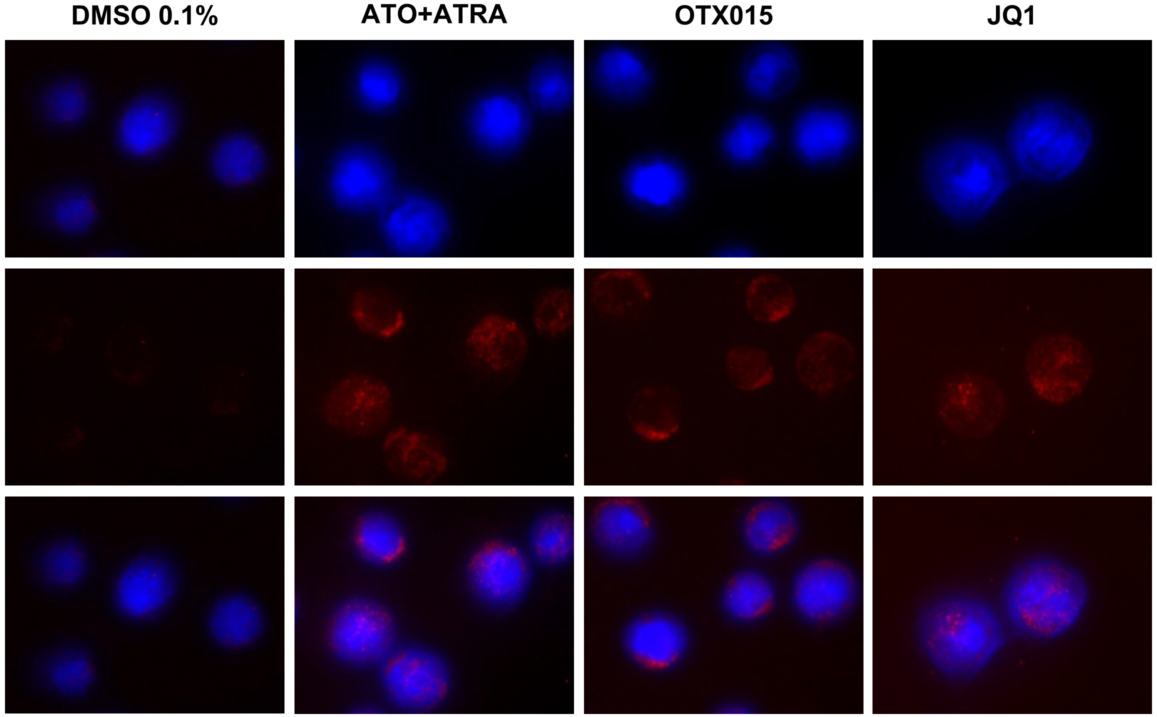

Supplementary Figure S4

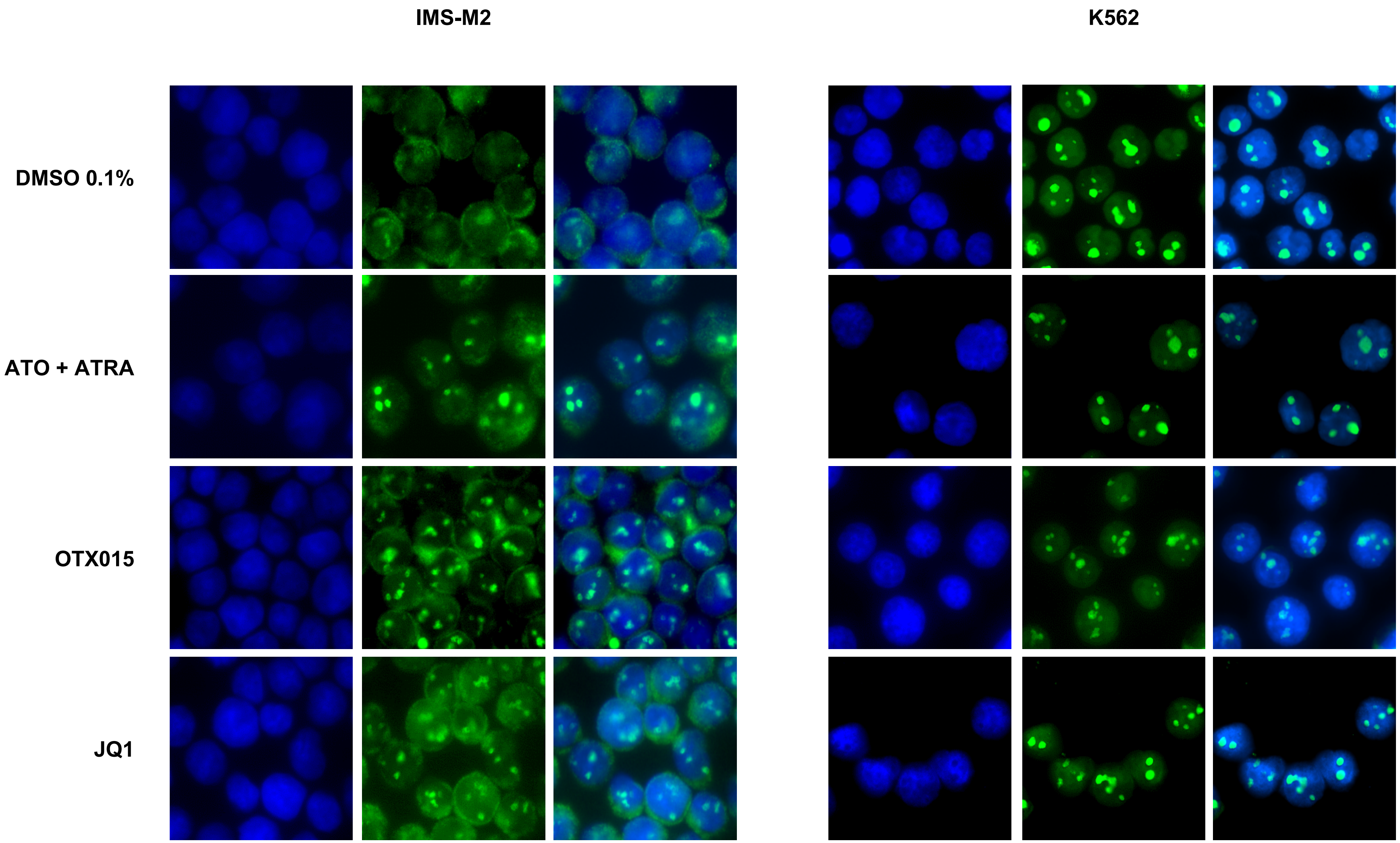

Supplementary Figure S5

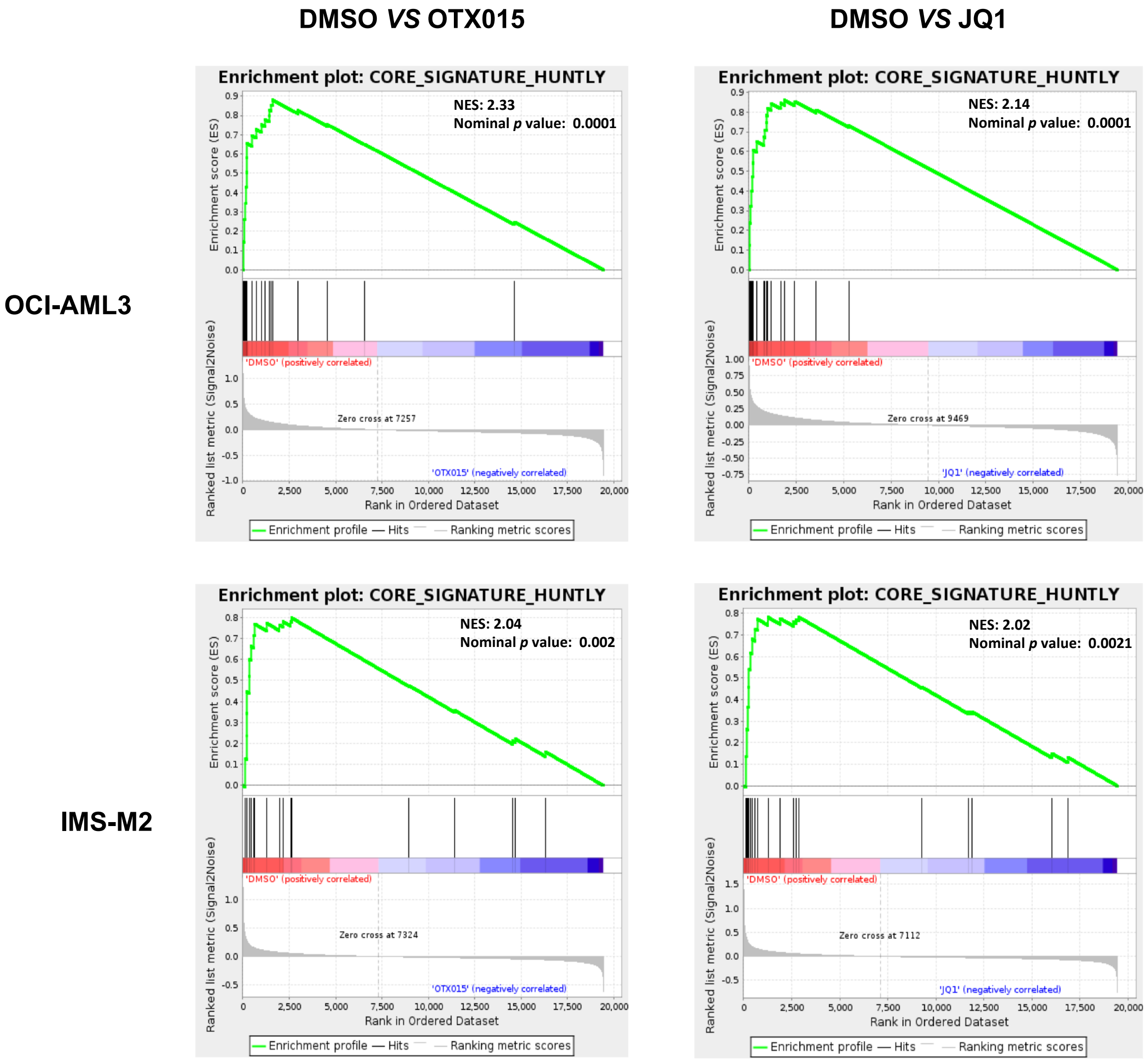

Supplementary Figure S6
